# Mechanical behavior of functionally zoned hybrid intracranial stent integrating laser-cut and braided architectures

**DOI:** 10.3389/fbioe.2026.1846079

**Published:** 2026-07-02

**Authors:** Lei Ma, Mingxuan Li, Maosong Chen, Dongfeng Wang, Zhenqiang Li, Gengfan Ye, Chuan Qiao, Zhongyou Li, Gen Lv, Chong Chen, Yang Wang, Daijun Shen, Ge Gao

**Affiliations:** 1 Department of Neurosurgery, Ningbo Medical Center Lihuili Hospital, Ningbo, Zhejiang, China; 2 Sichuan Province Biomechanical Engineering Laboratory, Sichuan University, Chengdu, China; 3 Department of Mechanical Science and Engineering, Chengdu, China; 4 Department of Biomedical Engineering, Faculty of Engineering, The Hong Kong Polytechnic University, Hong Kong, Hong Kong SAR, China; 5 Research Institute for Sports and Technology, The Hong Kong Polytechnic University, Hong Kong, Hong Kong SAR, China; 6 Chengdu Neurotrans Medical Devices Co., Ltd., Chengdu, Sichuan, China; 7 Department of Nephrology, Kidney Research Laboratory, West China Hospital of Sichuan University, Chengdu, China; 8 Department of Neurosurgery, People’s Hospital of Yanting, Chengdu, Sichuan, China; 9 Department of Neurosurgery, The First Affiliated Hospital of USTC, Division of Life Sciences and Medicine, University of Science and Technology of China, Hefei, Anhui, China

**Keywords:** braided stent, finite element analysis, flow diverter, laser-cut stent, mechanical properties

## Abstract

A novel functionally zoned hybrid intracranial stent is proposed to resolve the multifaceted mechanical limitations of existing designs by heterogeneously integrating a laser-cut anchoring segment with a braided section via interwoven filaments traversing precision-machined holes. Comprehensive finite element analysis and *in vitro* mechanical testing demonstrate that the optimal single-ended hybrid design simultaneously achieves five critical performance metrics: intermediate radial strength (2.89 N) between pure braided (2.12 N) and laser-cut (5.04 N) stents; comparable flexibility to braided stents (bending stiffness 0.00022 N/mm vs. 0.00023 N/mm); reduced foreshortening (18.1% vs. 22.2% and 22.0% for braided configurations); elimination of the end-constriction phenomenon through the laser-cut segment’s traction effect to ensure continuous wall apposition; and preserved structural integrity during 180° bending where pure laser-cut stents fractured at 90°. Acute implantation of braided stents in rabbit subclavian arteries indirectly validates the conformability and flow-diversion capability of the braided component, confirming that this functionally zoned heterogeneous integration strategy provides a mechanically comprehensive endovascular solution for complex wide-necked intracranial aneurysms.

## 1 Introduction

Intracranial aneurysms (IAs), detected in approximately 3% of the general population (Greving et al., 2014), carry significant mortality and morbidity upon rupture (Ziebart et al., 2024). While microsurgical clipping achieves high occlusion rates with low recurrence risk, endovascular treatment reduces procedural trauma and is preferred as the first-line option for most lesions (Molyneux, 2002; Muehlschlegel, 2018; Van Lanen et al., 2020). Since the advent of stent-assisted coiling in 2003 (Henkes et al., 2003), and culminating in the 2011 U.S. Food and Drug Administration approval of the Pipeline embolization device for large and giant aneurysms (U.S. Food and Drug Administration, 2011)—with clinical evidence demonstrating complete occlusion rates exceeding 95% at five-year follow-up (Becske et al., 2017)—the progressive maturation of flow diverter technology heralds a strategic paradigm shift from intra-aneurysmal embolization to hemodynamic reconstruction. This evolution underscores the increasingly critical role of intracranial stents in aneurysm treatment, now widely applied in the management of unruptured IAs and vascular stenosis (Zhang et al., 2019; Boisseau et al., 2023; Pielenz et al., 2025).

The mechanical performance of intracranial stents encompasses five critical metrics: radial strength, axial flexibility, wall apposition, foreshortening, and end-constriction. Radial strength denotes the capacity to resist external compression and maintain vascular patency; insufficient strength precipitates stent recoil and incomplete vessel dilation (Kumar and Bhatnagar, 2021). Axial flexibility reflects the ability to undergo longitudinal bending deformation; inadequate flexibility predisposes stents to kinking, fracture, or ovalization when navigating complex cerebrovascular geometry (Ebrahimi and Claus, 2007). Inadequate wall apposition leads to thrombus retention zones and elevated thromboembolic risk (Heller and Malek, 2011b), whereas foreshortening (defined as the percentage change in length from the compressed to the expanded state) impairs positioning accuracy and complete coverage of the target lesion (Cho et al., 2017). End-constriction, characterized by localized diameter reduction at stent ends resembling a constricted sleeve, arises from unconstrained filament sliding at free ends in braided stents. This inherent geometric instability compromises luminal patency at stent extremities, creating regions of incomplete wall apposition that predispose to thrombus formation and flow disturbance (Shanahan et al., 2017).

Based on manufacturing methodologies, intracranial stents are categorized into braided and laser-cut systems, each presenting distinct mechanical profiles and clinical limitations (Caton et al., 2021). Braided stents, fabricated from interwoven metallic filaments that permit relative sliding at crossing points, confer superior flexibility and wall apposition in tortuous vessels but exhibit inherently lower radial strength and pronounced foreshortening (typically 25%–45%) that complicates precise deployment and positioning (Cho et al., 2017). Conversely, laser-cut stents manufactured from seamless tubular constructs employ closed-cell architectures characterized by high radial strength and minimal foreshortening, yet their limited conformability impedes intimate wall apposition in curved anatomies, potentially causing strut kinking, end-flaring, or protrusion into the aneurysm neck that compromises flow diversion efficacy (Heller and Malek, 2011a; Maurer et al., 2023). Stent detachment, migration, or incomplete wall apposition resulting from suboptimal mechanical properties constitute major complications that compromise therapeutic efficacy and patient safety (Broadbent et al., 2003; Kim et al., 2008; Noh et al., 2023).

The increasing clinical demand for treating complex intracranial aneurysms in tortuous vasculature has highlighted a fundamental clinical paradox inherent to existing monolithic stent designs: In the small-caliber, highly curved cerebrovascular anatomy characteristic of intracranial aneurysms, current devices fail to simultaneously accommodate vascular tortuosity for optimal flow diversion and intimate wall apposition while ensuring secure distal fixation to prevent migration or detachment (Heller and Malek, 2011b; Cho et al., 2017). To address this unmet clinical need, this study proposes a novel functionally zoned hybrid stent design that integrates laser-cut anchoring segments with a braided section via an innovative interconnection mechanism wherein braided filaments traverse precision-machined holes. This heterogeneous architecture preserves the compliant braided mesh to ensure favorable wall apposition and flow-diversion capability, while strategically employing a high-stiffness laser-cut segment for distal anchoring and migration resistance, thereby resolving the fundamental trade-off between radial strength and flexibility that has long constrained neurointerventional stent design. We hypothesize that this hybrid configuration achieves balanced mechanical performance through functional zoning. Through FEA, *in vitro* mechanical testing, and animal experiments, we validated that this design achieves intermediate radial strength while maintaining braided-level flexibility, eliminates end-constriction, minimizes foreshortening, and ensures superior wall apposition compared to conventional stents.

## 2 Methodology

### 2.1 Novel functionally zoned hybrid stent design

**FIGURE 1:**
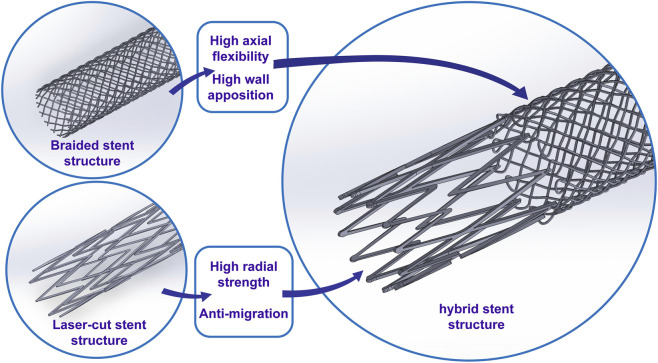
Schematic illustration of the functionally zoned hybrid stent design.

Building upon the preceding analysis, we present a functionally zoned hybrid stent concept (Figure 1). The device integrates laser-cut end segments with a braided section through an interconnection mechanism wherein braided filaments traverse precision-machined holes in the laser-cut portions, realizing a unified structure. This design synergistically combines the high radial strength and longitudinal dimensional stability of laser-cut architectures with the exceptional conformability and apposition characteristics of braided meshes. Strategically, this configuration enables firm distal fixation in the parent artery to resist dislodgement, accommodates vascular tortuosity for optimal hemodynamic modification, and ensures structural integrity through a connection interface that simplifies fabrication complexity.

### 2.2 Finite element analysis

**FIGURE 2:**
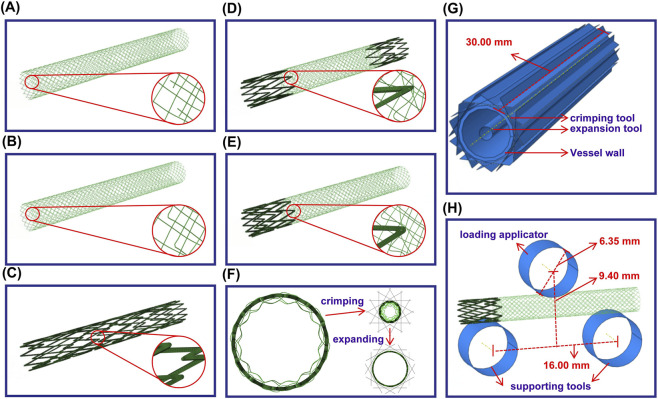
Finite element models and simulation configurations: **(A)** braided stent I, **(B)** braided stent II, **(C)** laser-cut stent, **(D)** hybrid stent I, and **(E)** hybrid stent II; **(F)** side view of hybrid stent II during the crimping-expansion sequence demonstrating intimate contact with the tools; and schematic illustrations of **(G)** stent crimping-expansion simulation and **(H)** three-point bending configuration.

To enable comparative benchmarking, five three-dimensional finite element models were constructed: a conventional laser-cut stent, braided stent I (featuring independent filaments without terminal interconnections, analogous to “open ends” designs (Shanahan et al., 2017; Zhao et al., 2021)), braided stent II (incorporating paired-end filament connections, equivalent to “looped ends” or “closed ends” configurations (Shanahan et al., 2017; Zhao et al., 2021)), hybrid stent I (laser-cut–braided–laser-cut architecture), and hybrid stent II (laser-cut–braided architecture) (Figure 2). Simulations of the crimping-expansion sequence and three-point bending mechanics were performed using Abaqus/Explicit 2025. The inclusion of braided stents I and II specifically isolates the effect of terminal connectivity on braided scaffold mechanics, providing insight into how such connections might influence hybrid stent integrity.

#### 2.2.1 Modeling and mesh generation

Stent dimensions were determined based on typical anatomical geometries of intracranial vasculature and aneurysms (Kim and Ko, 2014), informed by established stent design studies (Nishimura et al., 2019). The nominal specifications comprise a 3 mm diameter and 22 mm length.

The laser-cut stent and the laser-cut components of hybrid stents I and II employed a “W + M″ hybrid cell design with a nominal wall thickness of 0.1 mm (Figures 2C–E). Geometries were modeled in SolidWorks 2025 and imported into Hypermesh 2025 for discretization using hexahedral elements (four layers through the wall thickness). Localized mesh densification was applied to strut corners in crimping-expansion simulations, while uniform mesh distribution was used for three-point bending. The standalone laser-cut stent featured a repeating pattern of 2 cell types (length ratio: 2:3, total length: 22 mm). Hybrid stent I utilized a two-segment laser-cut structure (length ratio: 2:3, total length: 5 mm) with pre-drilled interconnection apertures (diameter: 0.04 mm). Hybrid stent II adopted an optimized three-segment configuration (short-to-long ratio: 7:11, total length: 5 mm) with pre-drilled apertures (diameter: 0.05 mm) to enhance radial support. The braided architectures of stents I/II and the braided segments of hybrid stents I/II (Figures 2A,B,D,E) comprised 32 interwoven filaments with a diameter of 0.02 mm. Using equation-driven curves in SolidWorks 2025, two fundamental helical trajectories were established according to Equation 1, where t_0_ and t_1_ denote the initial and final values of the parametric variable t defining the helical length. By fixing t_0_ and t_1_ while incrementing the phase angle by π/8 for successive filament pairs, the 2π circumferential arrangement of all 32 filaments was achieved. For braided stent II and hybrid stents I/II, interconnecting curves were manually constructed at the junctions to ensure coincident endpoints between paired filaments while maintaining the overall 22 mm length. These geometric curves were subsequently imported into Hypermesh 2025 and discretized using beam elements with controlled mesh density ensuring uniform global discretization.

$$\begin{array}{l} \begin{array}{l} \text{line }I=\left\{\begin{array}{l} x\left(t\right)=\left(1.5+0.013*\sin\left(16*t+\text{pi}/2\right)\right)*\cos\left(t\right)\;\\ y\left(t\right)=\left(1.5+0.013*\sin\left(16*t+\text{pi}/2\right)\right)*\sin\left(t\right)\\ z\left(t\right)=1.5*t \end{array}\right.\\ \text{line II}=\left\{\begin{array}{l} x\left(t\right)=\left(1.5+0.013*\sin\left(16*t+3*\text{pi}/2\right)\right)*\cos\left(t\right)\;\\ y\left(t\right)=-\left(1.5+0.013*\sin\left(16*t+3*\text{pi}/2\right)\right)*\sin\left(t\right)\\ z\left(t\right)=1.5*t \end{array}\right. \end{array}\\ t=\left\{\;\begin{array}{c} t_{0}\\ t_{1} \end{array}\right. \end{array}$$ (1)

In the structural design of hybrid stents, particular attention was devoted to the junction regions to ensure structural continuity as braided filaments traverse the laser-cut segments. To accommodate the braided filaments passing through the precision-machined holes, we implemented localized wall thickening at these interconnection zones (Figure 2F). Crucially, this localized thickening did not compromise the overall deformability of the stent. Taking hybrid stent II as an example, both the crimping-expansion and three-point bending simulations demonstrated intimate contact between the stent and the tooling surfaces (Figures 2F, 7E), thereby preserving computational accuracy while preventing mesh distortion.

As illustrated in Figure 2G, the crimping-expansion simulation employed a crimping assembly comprising 12 rectangular plates (3 mm × 30 mm) arranged cylindrically, alongside an expansion mandrel of 0.4 mm radius and 30 mm length. The vascular model, representative of human intracranial arteries, featured a length of 30 mm, inner diameter of 2.9 mm, and wall thickness of 0.2 mm. For three-point bending (Figure 2H), three parallel rigid cylinders with a diameter of 6.35 mm served as the loading applicator and static supports. All tools and vascular structures were modeled and discretized in Abaqus. The meshed stent models were imported into Abaqus where element types were assigned: S4R shell elements for tooling, C3D8 solid elements for the vessel, C3D8R solid elements (with hourglass control) for laser-cut components, and B31 beam elements for the braided architecture. To minimize mesh dependency, a mesh sensitivity study was conducted, requiring that the relative error in von Mises stress at corresponding critical locations between two successively refined mesh densities remain below 5%.

#### 2.2.2 Material properties and loading conditions

This study focuses on mechanical performance enhancement derived from novel structural architectures; accordingly, material parameters and contact definitions were appropriately simplified to facilitate computational efficiency. The stent was modeled as a Nitinol material using Auricchio–Taylor superelastic homogeneous isotropic material properties (Table 1) (Kleinstreuer et al., 2008; Kan et al., 2021), consistent with the experimental Nitinol prototypes employed in subsequent *in vitro* mechanical testing. To obtain discernible vascular deformation following stent deployment while fully accounting for intracranial vascular characteristics, the vessel wall was defined as a nearly incompressible isotropic two-parameter Mooney–Rivlin hyperelastic material (Table 1) (Leng et al., 2021). The expansion tool was set as an extremely high stiffness surface, whereas the crimping tools and three-point bending fixtures were modeled as rigid bodies (Wu et al., 2011; Grogan et al., 2012), with rigid body constraints applied via reference points for global motion control to render specific material properties inconsequential—an approach that serves as an alternative to assigning extremely high stiffness surfaces (Takashima et al., 2007; Gupta and Meena, 2023).

**TABLE 1:** Material properties of components used in this study. Nitinol superelastic parameters were adopted from Kleinstreuer et al. (2008). Vessel wall hyperelastic parameters were derived from human cerebral artery data (Leng et al., 2021). The tool was modeled as a rigid body with nominal steel properties.

Component	Density (g·cm^-3^)	Elastic modulus (MPa)	Poisson’s ratio	Material property
Stent	6.45	51,700 $\left(E_{A}\right)$	0.30 $\left(\nu_{A}/\nu_{M}\right)$	Auricchio–Taylor superelasticity: $E_{M}=47,800\text{ MPa}$ , $\varepsilon_{L}=0.063$ , $\sigma_{L}^{S}/\sigma_{L}^{E}=600/670\text{ MPa}$ , $\sigma_{U}^{S}/\sigma_{U}^{E}=288/254\text{ MPa}$ , $\sigma_{\text{CL}}^{S}=900\text{ MPa}$ , $T_{\text{ref}}=37\;{}^{\circ}C$
Vessel wall	1.20	—	—	Mooney–Rivlin hyperelasticity: $C_{10}=0.174\text{ MPa}$ , $C_{01}=1.88\text{ MPa}$ , $D_{1}=0.001\;{\text{MPa}}^{-1}$
Tool	7.98	200,000	0.30	—

Regarding contact definitions, hard contact was employed in the normal direction for all interactions. The self-contact of the laser-cut stent segment was defined as frictionless. For the braided architecture, inter-filament interactions at crossing points were realized through the Abaqus/Explicit general contact algorithm with beam cross-section assignment (circumscribed circle), enabling the B31 beam elements to engage in contact detection based on the outer boundary of their actual circular cross-sections and thereby accurately capturing inter-wire sliding behavior; the tangential behavior was governed by a penalty friction formulation with a coefficient of 0.2 (Fu et al., 2018; Yu et al., 2022). Contacts between the stent and tools/vessel likewise utilized a tangential penalty friction formulation with a coefficient of 0.2 (Zarins et al., 2004; Leng et al., 2018).

In the crimping-expansion simulation, the tooling configuration followed ASTM F3067-14 (ASTM International, 2014), consistent with multi-plate crimping protocols employed in comparable stent studies (Qiu et al., 2018); the sequence comprised four sequential analysis steps: crimping, release, expansion, and elastic recoil. Displacement-controlled boundary conditions were applied to the tooling to replicate the stent implantation procedure. During crimping, the stent was radially compressed by 1 mm; in the release step, the crimping plates were retracted radially to simulate stent deployment; subsequently, the expansion mandrel was inflated radially by 1.1 mm to achieve a final diameter of 3.0 mm (slightly exceeding the vessel inner diameter) to facilitate stent expansion; finally, the mandrel was retracted to allow elastic recoil of both stent and vessel. To prevent rigid body motion during crimping-expansion, appropriate nodes located at the mid-length plane along the stent axis were constrained against axial displacement. Specifically for hybrid stent II, nodal displacement control was implemented at the junction between laser-cut and braided segments due to differential axial elongation between the two structures during crimping: four selected nodes on the laser-cut component were subjected to controlled axial displacement, with the tool positioned axially in advance to accommodate this relative motion. For hybrid stent I, the higher radial stiffness of the laser-cut ends generates greater frictional interaction with the crimping tools compared to the braided segment; therefore, four nodes at each laser-cut end were prescribed a 2 mm axial displacement load to prevent mesh distortion caused by the braided section’s elongation being constrained by the rigid end segments. For three-point bending, tool dimensions and positioning followed ASTM F2606-08 standards (ASTM International, 2008). The loading applicator was subjected to a 1.75 mm vertical downward displacement within a single analysis step using a smooth amplitude curve, while the two support cylinders remained fixed. To mitigate localized mesh distortion in hybrid stents I and II, kinematic coupling was applied to the nodes on the inner surfaces of the pre-drilled apertures, enforcing shared displacement and load distribution to prevent adverse elemental deformation at the junctions.

Explicit dynamic simulations were performed for all five stent configurations under both crimping-expansion and three-point bending conditions. Analysis step durations, smooth amplitude loading curves, and local mass scaling parameters were carefully controlled to ensure quasi-static conditions throughout the simulations, maintaining kinetic energy below 5% of internal energy (De Beule et al., 2008; Grogan et al., 2012; Schiavone et al., 2014). Stress distribution contours were generated for both deformed and undeformed configurations. During the crimping-expansion sequence, the radial resisting force (F_radial_ (N)) and corresponding displacement values were recorded. In the crimping-expansion simulation, the radial force of the stent was defined as the sum of reaction forces exerted by the 12 crimping plates, as calculated in Equation 2:

$$F_{\text{radial}}=\sum_{i=1}^{12}F_{i}$$ (2)

where F_i_ (N) denotes the radial force component exerted by an individual crimping plate. For three-point bending, the reaction force and displacement of the loading applicator were recorded. The bending stiffness (K_bending_, N/mm) was determined from the linear region of the force-displacement curve according to Equation 3:

$$K_{\text{bending}}=\frac{△F}{△\delta }$$ (3)

where △F (N) represents the force variation within the linear segment of the load-displacement curve, and △δ (mm) denotes the corresponding displacement increment. The slope of this linear fit thus defines the three-point bending stiffness of the stent.

### 2.3 Stent fabrication and mechanical testing

**FIGURE 3:**
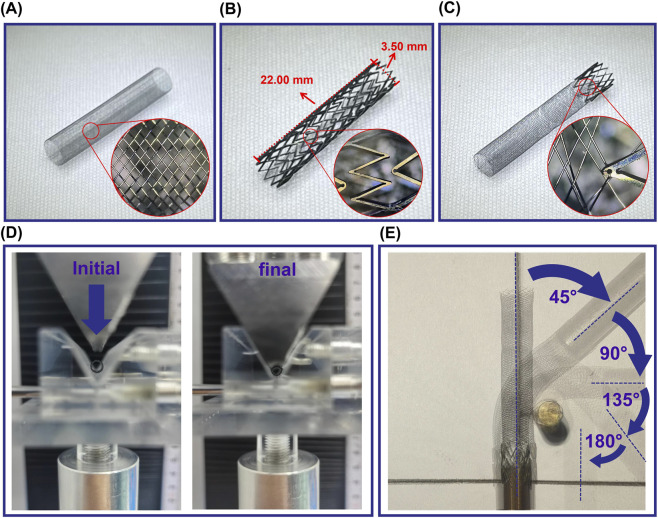
Photographs of fabricated stent prototypes: **(A)** braided stent I, **(B)** laser-cut stent, and **(C)** hybrid stent II. **(D)** Radial compression testing setup and **(E)** schematic illustration of the bending test.

Following finite element analysis, physical prototypes were fabricated for mechanical validation. Based on the simulation results, three stents—braided stent I, laser-cut stent, and hybrid stent II—were manufactured from Nitinol and subjected to radial strength and flexibility testing (Figures 3A–C), specifically to assess the mechanical performance enhancement of the hybrid design. The radial force test was performed with reference to YY/T 0663.2-2024 (China Food and Drug Administration, 2024) to standardize the extraction of radial supporting force, whereas the bending test served as a proof-of-concept validation, with flexibility assessed by observing stent deformation under prescribed bending angles.

Retain the finite element models of braided stent I and hybrid stent II as design references, and homogenize the laser-cut stent pattern: repeat the short-segment length of hybrid stent II 14 times along the longitudinal axis to obtain a 22 mm laser-cut stent with consistent structural parameters (hereinafter, both the laser-cut stent model in finite element simulations and the physically fabricated laser-cut stent are collectively referred to as the “laser-cut stent”). Laser cutting was performed on Nitinol tubing with an inner diameter of 3.5 mm and wall thickness of 0.1 mm. The braided structures of both braided stent I and hybrid stent II were fabricated using 0.03 mm diameter Nitinol filaments on a 32-carrier horizontal braiding machine, with an inner diameter of 3.5 mm and a PPI (picks per inch) of 155. Braided stent I was braided directly and subsequently truncated at 22 mm. For hybrid stent II, Nitinol filaments were first threaded through the pre-drilled apertures of the laser-cut segment before braiding, and likewise truncated at 22 mm, thereby ensuring uniform structural geometry, length, and inner diameter across all three stent prototypes.

For radial strength testing, the three stents were individually positioned on a mechanical testing machine (Figure 3D). A compression platen was advanced at a uniform rate to compress each stent radially by 1 mm, with displacement and reaction forces recorded continuously throughout the loading sequence. For the bending test (Figure 3E), a cylindrical metal rod (diameter: 2.7 mm) was secured to the platform at coordinates (8 mm, 3 mm) relative to the reference axes, oriented perpendicular to the surface to serve as the bending fulcrum. The three stents were individually fixed at the origin using a 3.5 mm diameter metal rod. Bending deformation was documented photographically at 45° intervals from 0° to 180° to characterize the flexibility behavior.

### 2.4 Animal experiments

**FIGURE 4:**
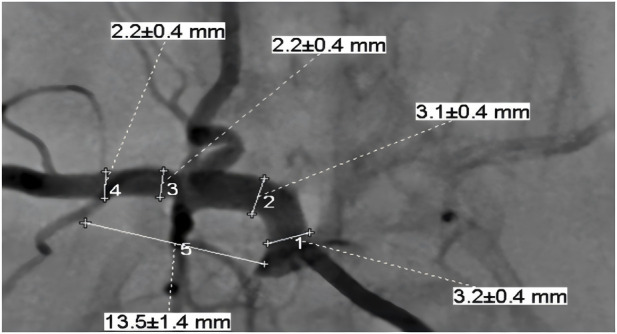
Angiographic visualization and dimensional measurements of the rabbit subclavian artery (diameter: 2.5–3.5 mm), employed as a surrogate model for human intracranial vasculature.

Consistent with the functionally zoned design strategy proposed herein, the shorter laser-cut segment serves as the distal anchoring and radial-force enhancement zone, whereas the longer braided segment functions as the conformability and flow-diversion zone—meaning the braided section constitutes the primary therapeutic functional region for aneurysm treatment. To validate the therapeutic efficacy of the braided stent architecture, the rabbit *in vivo* experiments were designed to evaluate two specific challenges of the braided component: the acute challenge—deliverability through 90° tortuosity and wall apposition at the aneurysm neck; and the chronic challenge—maintenance of radial support and flow-diversion efficacy over a 12-month period under hemodynamic stresses. Given the anatomical challenges and small caliber of intracranial arteries, the rabbit subclavian artery was selected as a surrogate model for human intracranial vasculature. The subclavian artery exhibits a diameter of approximately 2.5–3.5 mm and a curvature of approximately 90° (Figure 4), comparable to human intracranial vessels and adequately simulating tortuous aneurysmal anatomy.

Three conventional-grade adult rabbits were used. They were quarantined for 7 days with *ad libitum* access to standard laboratory rabbit chow and drinking water. Following the quarantine period, the animals were fasted for 8–12 h and water-restricted for 2–4 h prior to surgery. General anesthesia was induced via intramuscular injection of Zoletil 50 at a dosage of 3–8 mg/kg. Respiratory rate, heart rate, and corneal reflexes were monitored every 15 min, with supplemental anesthetic administered as needed to maintain an appropriate depth of anesthesia. The surgical site was shaved and disinfected with iodophor. The right common carotid artery was exposed, and the proximal blood flow was temporarily occluded. Porcine pancreatic elastase (100 U/mL) was infused into the arterial lumen and incubated for 15–20 min under balloon occlusion to digest the intima and media. The proximal occlusion was then released, and the distal vessel was ligated. The incision was closed in layers, and the animals were allowed to recover. After 28 days of housing, stent implantation was performed. Stent deformation during deployment was recorded in real time to assess procedural difficulty. The experimental endpoint was set at 12 months post-operation. Prior to euthanasia, follow-up evaluation was performed to assess aneurysm treatment outcomes. Following approval by the Institutional Ethics Committee, euthanasia was conducted via rapid intravenous injection of KCl solution (10 mL: 1 g) at 200 mg/kg through the marginal ear vein while under deep anesthesia. Following injection, a veterinarian confirmed death by cessation of heartbeat and respiration, fixed and dilated pupils, and absence of corneal and nociceptive reflexes prior to tissue collection.

Finite element analysis results and *in vitro* experimental data were integrated and evaluated across three mechanical performance dimensions: radial strength, flexibility, and wall apposition (including foreshortening and end-constriction). *In vivo* animal experimental results are presented separately.

## 3 Results

### 3.1 Radial strength

**FIGURE 5:**
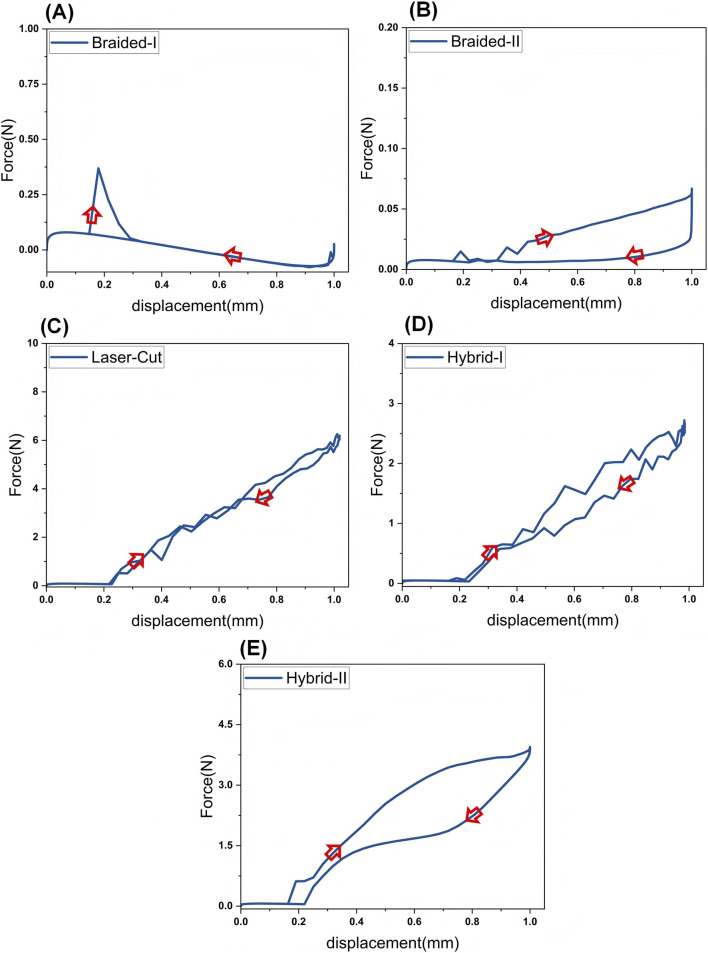
Radial force-displacement curves of stents under radial compression (red arrows indicate the loading direction): **(A)** braided stent I, **(B)** braided stent II, **(C)** laser-cut stent, **(D)** hybrid stent I, and **(E)** hybrid stent II.

**FIGURE 6:**
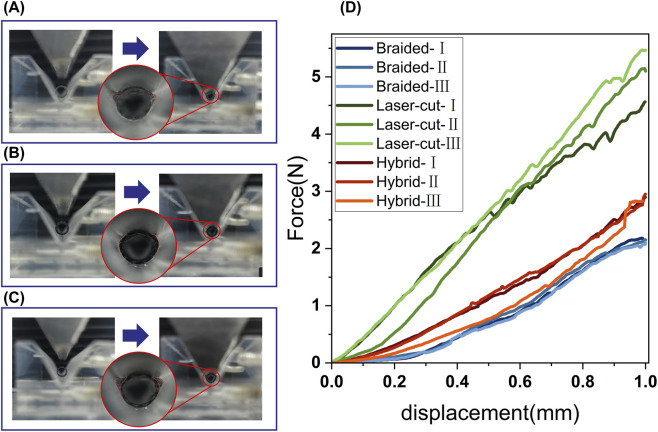
Radial compression testing of stents: **(A–C)** deformation comparison before and after testing for **(A)** braided stent I, **(B)** laser-cut stent, and **(C)** hybrid stent II, showing more pronounced deformation in the braided structure; **(D)** radial force-displacement curves of the three stents. Under 1 mm radial compression, the hybrid stent exhibited an intermediate radial force of 2.89 N, between that of the braided stent (2.12 N) and the laser-cut stent (5.04 N), representing a 36.3% increase compared to the braided stent.

Both FEA and experimental testing demonstrated consistent radial strength hierarchies across stent configurations. Under 1 mm compression, the laser-cut stent achieved the highest FEA-predicted radial force (6.19 N), whereas braided stents showed the lowest values (0.01 N and 0.06 N for braided stents I and II, respectively). Hybrid stent II achieved an intermediate FEA value (3.94 N), representing a 48.1% increase over hybrid stent I (2.66 N). Experimentally, hybrid stent II exhibited a radial force of 2.89 N—higher than the braided stent (2.12 N) but lower than the laser-cut stent (5.04 N)—confirming that the integrated laser-cut segment effectively enhances distal anchoring capability without excessive rigidity (Figures 5, 6).

### 3.2 Flexibility

**FIGURE 7:**
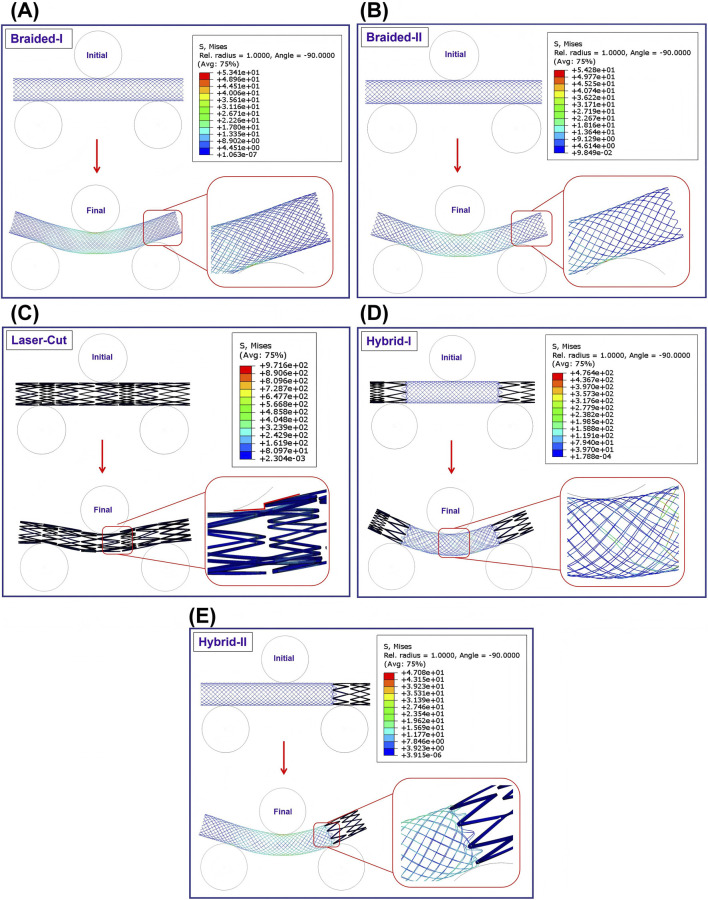
Stress distribution contours of stents under three-point bending (magnified views of regions indicated by red boxes): **(A)** braided stent I, **(B)** braided stent II, **(C)** laser-cut stent, **(D)** hybrid stent I, and **(E)** hybrid stent II. Both **(A)** and **(B)** exhibited coordinated deformation and uniform stress distributions without end-constriction. **(C)** The laser-cut stent demonstrated ovalization at the bending region with severe wall discontinuity (as indicated by the red line). In **(D)**, the braided segment of hybrid stent I exhibited localized stress concentration, whereas **(E)** hybrid stent II achieved a uniform stress distribution; in both configurations, the laser-cut segments underwent no significant deformation, with stress concentrating primarily at the junctions between the laser-cut and braided sections.

Three-point bending simulations revealed distinct stress distribution patterns across configurations (Figure 7). Both braided stent I and braided stent II exhibited uniform stress distributions, with peak von Mises stresses of 53.4 MPa and 54.3 MPa, respectively. The laser-cut stent demonstrated severe stress concentration at strut junctions (peak stress: 971.6 MPa) with ovalization and localized collapse, confirming compromised geometric stability under bending. In contrast, hybrid stent I exhibited localized non-uniform stress distribution in the braided segment, with localized stresses reaching up to 476.4 MPa and peak stresses exceeding 100.0 MPa at the junction. Hybrid stent II, however, achieved an overall uniform stress distribution in the braided segment, with peak stresses of 47.1 MPa localized at the junction. The laser-cut segments in both hybrid configurations provided radial geometric stability, constraining the braided sections without undergoing significant deformation themselves.

**FIGURE 8:**
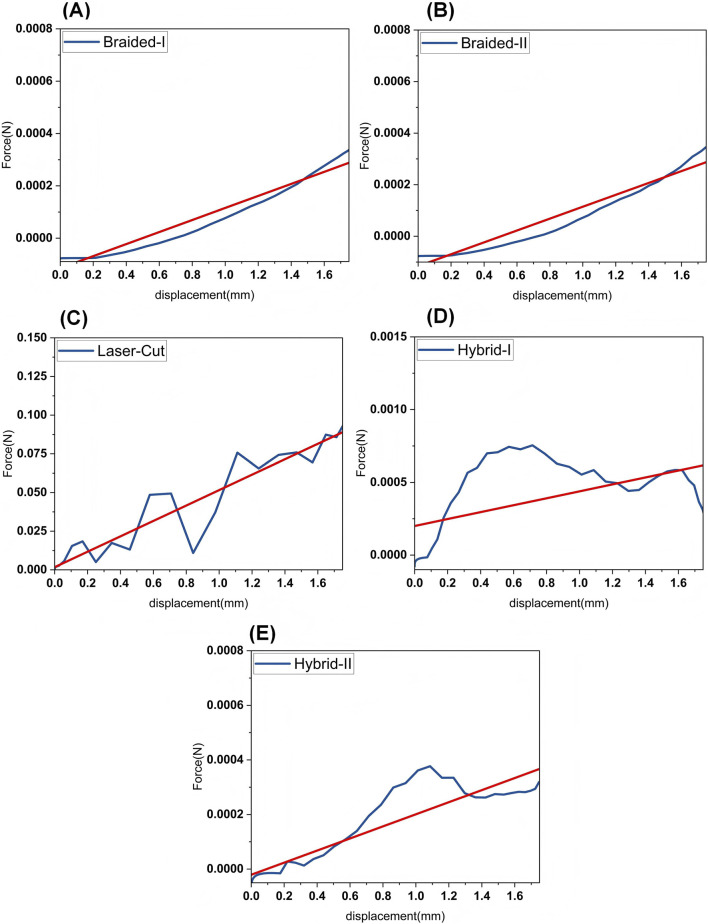
Vertical force-displacement curves of the loading applicator during three-point bending simulations: **(A)** braided stent I, **(B)** braided stent II, **(C)** laser-cut stent, **(D)** hybrid stent I, and **(E)** hybrid stent II.

Three-point bending stiffness values (derived from force-displacement curves, Figure 8) revealed that the laser-cut stent exhibited exceptionally high rigidity (0.04982 N/mm), exceeding other configurations by approximately two orders of magnitude. In contrast, hybrid stents I and II demonstrated bending stiffness values (0.00024 N/mm and 0.00022 N/mm, respectively) comparable to those of braided stents I and II (0.00023 N/mm and 0.00023 N/mm, respectively). These results confirm that both hybrid configurations successfully inherited the favorable axial flexibility characteristics of braided stents.

**FIGURE 9:**
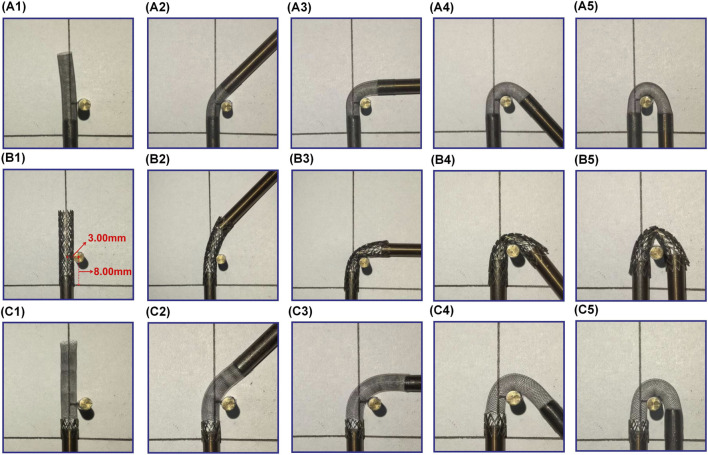
End-constrained bending deformation at 45° intervals from 0° to 180°: **(A1–A5)** braided stent I, **(B1–B5)** laser-cut stent, and **(C1–C5)** hybrid stent II.

Experimental bending tests validated the simulation findings (Figure 9). Braided stent I and hybrid stent II both exhibited smooth deformation from 0° to 180° while maintaining cross-sectional stability and structural integrity, with hybrid stent II demonstrating robust interconnection retention without displacement. Conversely, the laser-cut stent underwent catastrophic failure: pronounced deformation initiated at 45°, followed by severe kinking, ovalization, and structural fracture between 90° and 135°, ultimately resulting in dislodgement at 180°. These results confirm that while braided architectures accommodate extreme bending, laser-cut structures exhibit poor conformability with failure at 90°, and the hybrid design successfully preserves braided flexibility while ensuring interfacial structural continuity.

### 3.3 Wall apposition, foreshortening and end-constriction

**FIGURE 10:**
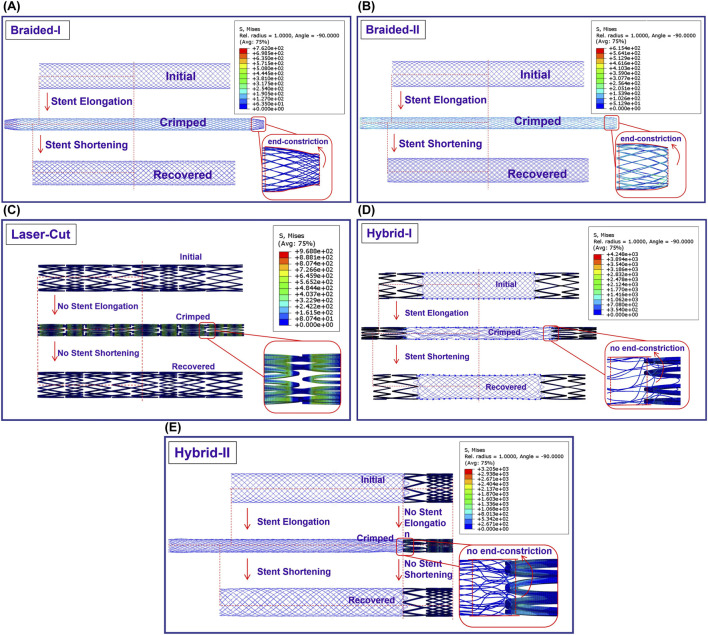
Stress distribution contours of stents at initial, crimped, and elastic recoil states (magnified views of regions indicated by red boxes): **(A)** braided stent I, **(B)** braided stent II, **(C)** laser-cut stent, **(D)** hybrid stent I, and **(E)** hybrid stent II. Both braided configurations exhibited end-constriction and axial shortening, with braided stent I showing more pronounced end-constriction and gradient stress decay, whereas braided stent II demonstrated stress concentration at terminal interconnections. **(C)** The laser-cut stent maintained dimensional stability but exhibited significant stress concentration at strut corners. In **(D)** and **(E)**, both hybrid configurations showed moderate axial shortening with stress concentrating at laser-cut strut corners, braided filaments, and surrounding apertures at the junctions.

Crimping-expansion simulations revealed distinct deformation behaviors (Figure 10). Laser-cut structures exhibited stress concentration at strut corners, whereas braided architectures demonstrated lower stress distributions attributable to inter-filament sliding, both braided stents exhibited uniform stress distributions. Throughout the crimping-expansion sequence, hybrid stent II—featuring a single-ended laser-cut configuration—did not excessively constrain the braided segment, yielding coordinated overall deformation. This indicates that hybrid stent II, by virtue of its predominantly braided composition (5/22 laser-cut), exhibits reduced fatigue susceptibility. Hybrid stent I exhibited constrained deformation characteristics: during crimping, the laser-cut ends elongated the braided segment by 4 mm, and in this process the braided filaments at both ends were unable to slide freely, resulting in filament disorganization and structural entanglement (Figure 10D), which may predispose the stent to structural failure or material fatigue; prior to the expansion phase, as the crimping tools gradually retracted their radial constraint, the laser-cut ends preferentially recoiled and expanded radially, driving deformation at the braided junctions and producing a non-uniform diameter profile with flared ends and a constricted mid-section, thereby potentially increasing procedural complexity during clinical deployment.

Quantitative foreshortening analysis revealed significant differences among configurations: braided stent I (22.2%), braided stent II (22.0%), pure laser-cut stent (0%), hybrid stent I (10.0%), and the hybrid stent II (18.1%). Owing to the terminal filament interconnection in braided stent II, its foreshortening was reduced by 0.2% compared to braided stent I, with markedly diminished end-constriction following crimping (Figures 10A,B). Both hybrid stents exhibited substantially reduced foreshortening relative to the braided stents.

**FIGURE 11:**
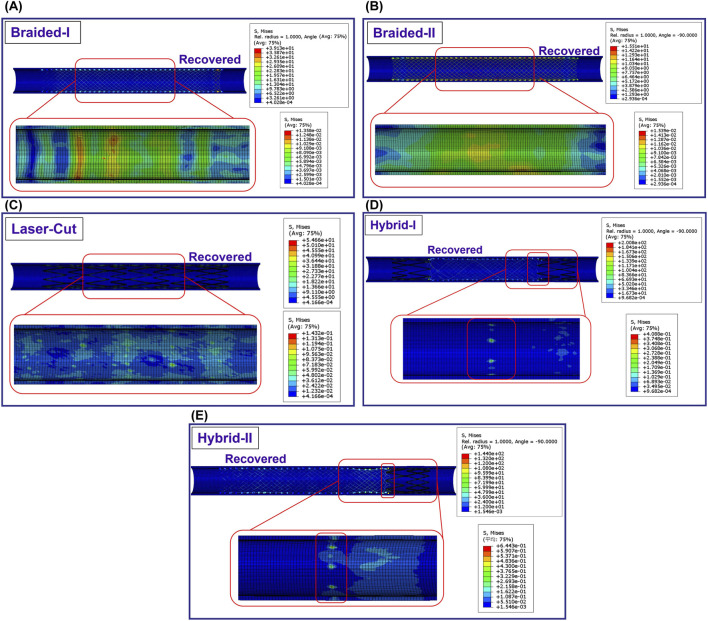
Vascular stress distribution contours following stent deployment and elastic recoil (magnified views of regions indicated by red boxes): **(A)** braided stent I, **(B)** braided stent II, **(C)** laser-cut stent, **(D)** hybrid stent I, and **(E)** hybrid stent II. **(A)** and **(B)** Both braided configurations exhibited uniform vascular stress distributions decreasing from the mid-segment toward both ends. **(C)** The laser-cut stent induced localized high stress and deformation at vessel wall regions corresponding to strut corner contacts. **(D)** and **(E)** Both hybrid stent I and hybrid stent II demonstrated relatively uniform vascular stress distributions in the braided segments corresponding to the vessel wall, with stress concentration primarily at the vessel wall of the junction between the laser-cut and braided segments, indirectly corroborating the distal anchoring function of the laser-cut segment on the braided segment.

Vascular stress analysis following deployment revealed distinct wall-stent interaction patterns (Figure 11). Braided stents I and II both exhibited uniform vascular wall stress distributions (1.0 × 10^−2^ MPa); braided stent II exhibited end-constriction due to plastic deformation at filament interconnections, with vascular wall stress demonstrating a gradual decrease toward the ends. The laser-cut stent induced higher mean vascular wall stress (3.5 × 10^−2^ MPa), with localized vascular wall stress values up to 4.5 × 10^−2^ MPa at corresponding strut corners. In hybrid stents I and II, high vascular wall stress (2.0 × 10^−1^ MPa) appeared at the junctions corresponding to the vessel wall; the laser-cut segments of both hybrid stents exhibited vascular wall stress comparable to that of the laser-cut stent (3.0 × 10^−2^ MPa and 4.0 × 10^−2^ MPa), and the braided segments exhibited vascular wall stress comparable to that of the braided stents (8.0 × 10^−3^ MPa and 1.0 × 10^−2^ MPa).

Regarding end-constriction, this phenomenon was exclusively observed in braided architectures: both braided stents exhibited end-constriction during the crimping-expansion sequence (Figures 10A,B); compared with braided stent I, braided stent II mitigated post-crimping end-constriction through terminal filament interconnection, yet slight end-constriction developed following stent release due to plastic deformation at the interconnections, and no end-constriction was observed in three-point bending simulations. Notably, both hybrid configurations completely eliminated end-constriction at the junctions (Figures 7D,E, 10D,E). The laser-cut segments possessed high radial stiffness, mechanically constraining the braided filaments through a traction effect to prevent the relative filament sliding that causes end-constriction in conventional braided architectures, thereby maintaining uniform luminal diameter throughout the stent length.

### 3.4 Animal experimental results

**FIGURE 12:**
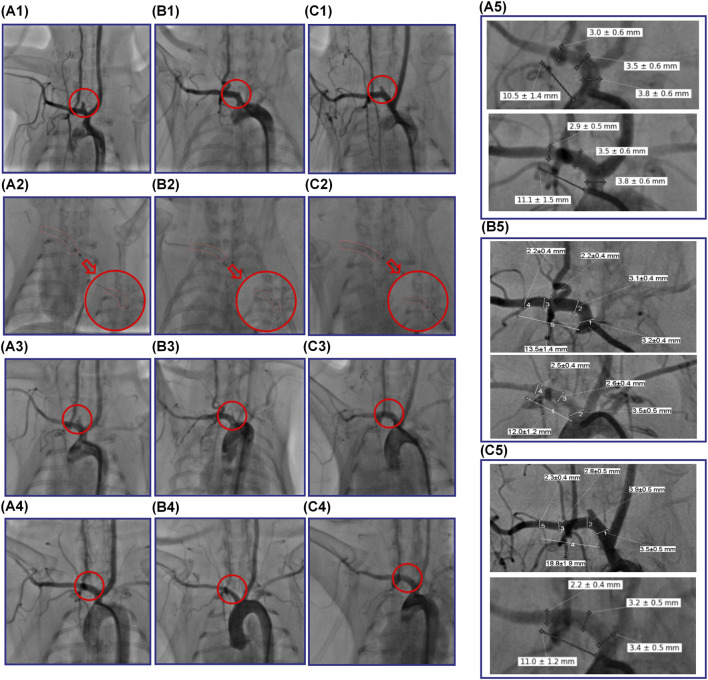
Stent deformation and angiographic assessment results from the rabbit subclavian artery aneurysm stenting study (aneurysm locations indicated by red circles): **(A1–C1)** Preoperative angiographic visualization of artificial subclavian artery aneurysms; **(A2–C2)** Immediate post-deployment views showing the braided stent conforming to vascular curvature with approximately 90° bending (within the red circles indicated by arrows); **(A3–C3)** Angiography immediately following 72-filament braided stent deployment, demonstrating blood flow guidance at the aneurysm site; **(A4–C4)** One-year follow-up angiography demonstrating complete aneurysm healing; and **(A5–C5)** Comparative dimensional measurements of the rabbit subclavian artery pre- and post-operation, the upper images represent vascular dimensional measurements post-operation, while the lower images represent measurements at 1-year follow-up.

One-year follow-up angiography confirmed complete aneurysm healing in all three rabbits (Figure 12), validating that braided stents satisfy fundamental mechanical requirements including successful deployment through 90° tortuosity, adequate wall apposition, and sustained 12-month radial support. However, real-time fluoroscopy revealed immediate axial foreshortening necessitating precise positioning adjustments, confirming the technical challenges of length reduction in tortuous vasculature. These findings underscore the clinical necessity of hybrid stent II, which preserves braided flexibility and flow diversion capability while reducing foreshortening to 18.1%, thereby mitigating deployment difficulties associated with pure braided configurations.

## 4 Discussions

### 4.1 Comparison with conventional laser-cut and braided stents

The mechanical performance trade-offs between laser-cut and braided stents have been well documented in comparative studies. Cho et al. (2017) demonstrated that laser-cut stents (Neuroform and Enterprise) exhibit minimal foreshortening but inherently lower flexibility, whereas braided stents (LEO and LVIS) undergo significant length reduction during deployment (typically 22%–25%) while offering superior conformability. Our FEA and experimental results quantitatively reproduce this dichotomy: the laser-cut stent achieved the highest FEA-predicted radial force (6.19 N) and bending stiffness (0.04982 N/mm), exceeding braided configurations by approximately two orders of magnitude in stiffness. Conversely, braided stents I and II exhibited the lowest radial forces (0.01 N and 0.06 N, respectively) and bending stiffness values (0.00023 N/mm for both), accompanied by pronounced foreshortening (22.2% and 22.0%) and end-constriction during crimping-expansion.

Zhang et al. (2021) conducted a meta-analysis of 4,373 patients revealing that laser-cut stents offer higher deployment success rates (98.69% vs. 97.07%) but are associated with increased permanent morbidity compared to braided stents (2.92% vs. 1.58%). These clinical findings align with our mechanical observations: the laser-cut stent achieved the highest FEA-predicted radial force (6.19 N) and experimental radial force (5.04 N), preventing migration but increasing the risk of vessel straightening and incomplete apposition in tortuous anatomy (Izar et al., 2011; Giraldo et al., 2023; Maurer et al., 2023), whereas braided stents exhibited the lowest FEA-predicted radial forces (0.01 N and 0.06 N) with an experimental anchoring force of 2.12 N, facilitating navigation but compromising distal fixation. Our hybrid stent II achieved an intermediate experimental radial force of 2.89 N (FEA: 3.94 N), and our functionally zoned approach resolves this dichotomy by providing laser-cut-level anchoring at the distal end (preventing migration) while preserving braided-level flexibility in the mid-section (accommodating tortuosity).

The end-constriction phenomenon was observed in both braided stent models during crimping-expansion (Figures 10A,B), manifesting as localized diameter reduction resembling a constricted sleeve. This phenomenon may lead to incomplete wall apposition and flow disturbance at the stent ends after deployment. Consistent with Shanahan et al. (2017), who demonstrated that open-ended braided stents exhibit significant end-constriction under compression compared to looped-end designs, our results confirm that braided stent I (open ends) exhibited more severe end-constriction than braided stent II (looped ends). Specifically, braided stent II showed stress concentration primarily at terminal interconnections following crimping-expansion, indicating that terminal connectivity enhances structural continuity but cannot fully eliminate geometric instability under extreme compression. Zhao et al. (2021) further elucidated that strong end constraint (closed looped ends) is essential for maintaining radial compression performance in braided stents, which aligns with our observation that braided stent II achieved 0.05 N greater radial strength than braided stent I (0.06 N vs. 0.01 N in FEA) through enhanced filament constraint. Building upon these findings, our hybrid design effectively eliminated end-constriction (Figures 7E, 10E) by introducing a rigid laser-cut segment that provides mechanical constraint far exceeding that of looped-end designs. The high radial stiffness of the laser-cut component (5.04 N experimentally) mechanically constrained the braided filaments through a traction effect, preventing the relative filament sliding that causes end-constriction in conventional braided architectures. This functional zoning strategy maintains uniform luminal diameter and ensures continuous wall apposition throughout the stent length, addressing the inherent geometric instability of open-ended braided stents identified by Shanahan et al. while overcoming the plastic deformation at terminal interconnections observed in looped-end designs.

### 4.2 Hemodynamic environment, functional zoning strategy, and clinical implications

The rabbit common carotid artery elastase-induced aneurysm model is a bifurcation-type artificial aneurysm located at the origin of the right common carotid artery. Due to the unique anatomical configuration at this site, the implanted stent is subjected to specific hemodynamic stresses. Zeng et al. (2011) systematically mapped the hemodynamic profile of this model, demonstrating Reynolds numbers of 200–400, peak systolic wall shear stress (WSS) in the parent artery (common carotid artery) of 2.16 ± 0.93 Pa, and aneurysm sac WSS averaging 0.49 ± 0.62 Pa with minimal WSS (<0.1 Pa) at the dome—features that closely mirror human intracranial aneurysms (Zeng et al., 2011). In this milieu, the stent is subjected not only to hemodynamic loads but also to persistent cyclic mechanical interaction with the vessel wall, encompassing four principal categories: pulsatile WSS acting directly on stent strut surfaces; cyclic drag forces normal to the stent mesh generated by blood-flow redirection; cyclic bending imposed by the curved and tapered course of the parent artery (proximal diameter 4.01 mm, distal diameter 3.26 mm) (Zeng et al., 2011); and radial compressive force arising from the combined action of vascular elastic recoil and pulsatile blood pressure.

The clinical efficacy of flow diversion depends critically on two competing requirements: sufficient metal coverage to disrupt intra-aneurysmal flow and adequate flexibility to achieve favorable wall apposition, alongside sufficient mechanical stability to withstand the aforementioned stresses without migration or geometric distortion. Cebral et al. (2014) showed that flow-diverting stents markedly reduce intra-aneurysmal WSS and disrupt flow patterns, creating hemodynamic conditions that favor slow-flow-induced thrombosis in rabbit models (Cebral et al., 2014). The clinical success of the Pipeline Embolization Device—achieving complete occlusion rates exceeding 95% at five-year follow-up (Becske et al., 2017)—directly validates the therapeutic effectiveness of braided constructs. However, high metal coverage alone cannot ensure therapeutic success; if the stent undergoes migration or fails to conform to the parent artery while simultaneously sustaining the aforementioned drag forces and bending strain, therapeutic efficacy is substantially compromised. Kühn et al. (2017) reported that migrated Pipeline devices required secondary laser-cut stent fixation, highlighting the clinical consequence of inadequate distal anchoring under hemodynamic drag forces (Kühn et al., 2017). Similarly, incomplete wall apposition creates thrombus retention zones and elevates thromboembolic risk, as demonstrated by Heller et al. (2013), who correlated incomplete stent apposition with delayed ischemic events in Enterprise stent-assisted coiling (Heller et al., 2013).

The concept of functional zoning—assigning distinct mechanical roles to specific stent segments—emerges as a rational strategy to resolve these competing demands. While prior studies have explored hybrid cell configurations within a single manufacturing methodology (e.g., the Neuroform Atlas proximal closed-cell/distal open-cell design (Hanaoka et al., 2020; Arslan et al., 2021)), this study represents the first investigation of heterogeneous structure integration combining laser-cut and braided architectures. Our hybrid stent II operationalizes this concept by designating the laser-cut segment as the distal anchoring zone and the braided segment as the flow-diversion zone: the laser-cut segment provides sufficient radial strength to resist vascular elastic recoil and maintain luminal patency, while simultaneously offering distal anchoring to resist migration under cyclic drag forces; the braided mid-section preserves the metal-coverage-dependent flow-diversion capability—we have achieved favorable therapeutic outcomes with the 72-filament braided stent in the rabbit common carotid artery aneurysm model (Figure 12), indirectly corroborating the therapeutic role of the braided segment in the hybrid construct. In clinical practice, these competing demands have manifested as distinct failure modes in monolithic designs: closed-cell laser-cut stents (e.g., Enterprise) deployed in tortuous intracranial vessels exhibit a high prevalence of incomplete stent apposition (ISA) and stent kinking—Heller and Malek (2011) detected ISA in 19 of 39 patients (49%) undergoing Enterprise stent-assisted coiling, with vessel curvature identified as an independent predictor (Heller and Malek, 2011b), while Tsuruta et al. (Tsuruta et al., 2013) further documented stent kinking in 7 cases and luminal flattening at the carotid siphon using CT. These geometric instabilities predispose to thrombus retention and delayed ischemic events (Heller et al., 2013). Conversely, braided stents suffer from excessive foreshortening (25%–45%) that complicates precise deployment and risks incomplete aneurysm-neck coverage (Cho et al., 2017), while end-constriction at free ends creates flow disturbance and thrombus formation (Shanahan et al., 2017). By eliminating end-constriction through the laser-cut segment’s traction effect, the hybrid design ensures uniform luminal geometry and continuous wall apposition, thereby mitigating the thromboembolic risks associated with incomplete apposition. This “semi-open” configuration (single laser-cut end) permits the braided segment to expand and contract freely, avoiding the excessive foreshortening observed when both ends are rigidly constrained, while reducing foreshortening to 18.1%—a critical advantage in tortuous intracranial vessels where excessive shortening may lead to incomplete aneurysm neck coverage or parent vessel injury (Cho et al., 2017). Thus, the hybrid design simultaneously addresses the acute hemodynamic stresses (WSS, drag, bending) and the chronic mechanical challenges (migration, foreshortening, end-constriction) inherent to flow-diverter therapy in complex intracranial aneurysms.

### 4.3 Interface design and structural integrity

The interconnection mechanism—braided filaments traversing precision-drilled apertures in the laser-cut segment—proved critical for maintaining structural continuity. First, braided stent II, through its looped-end configuration, significantly enhanced structural integrity compared to braided stent I, with a 0.05 N increase in radial strength and a uniform stress distribution, providing compelling evidence for the feasibility of employing looped-end braided filaments traversing the laser-cut segments in the hybrid design. In hybrid configurations, this interconnection design enabled cooperative deformation between segments. During expansion, the radial dilation of the laser-cut segment generated traction forces that pulled the braided section into apposition, thereby eliminating the end-constriction characteristic of standalone braided stents. However, stress analysis revealed elevated stress concentrations at the junction (up to 320.5 MPa in hybrid stent II), suggesting the need for geometric optimization—such as increasing aperture diameters or implementing filleted edges—to mitigate fatigue risks under pulsatile hemodynamics. In stark contrast to pure laser-cut stents—which exhibited severe kinking and ovalization at 90° bending (Figure 9B)—hybrid stent II maintained structural integrity throughout the full 180° bending test (Figure 9C), validating that the hybrid design inherits the excellent flexibility characteristic of braided stents. This favorable flexibility is essential for navigating tortuous intracranial vasculature, where conventional laser-cut stents frequently fail (Ebrahimi and Claus, 2007; Heller et al., 2013).

### 4.4 Study limitations and future directions

Several limitations should be acknowledged. First, the current finite element simulations employ a two-parameter Mooney–Rivlin hyperelastic model for the vessel wall. While this formulation accurately reproduces the nonlinear response of cerebral arteries, it remains homogeneous and isotropic, thereby neglecting patient-specific anatomical variations, residual stresses, and collagen fiber-induced anisotropy—all of which may substantially affect stent–vessel interactions and wall apposition predictions in tortuous segments. Second, the study is confined to quasi-static crimping–expansion and three-point bending simulations, with no pulsatile fatigue simulation or accelerated bench testing performed. The *in vitro* bending test served merely as a proof-of-concept experiment; quantitative measurement of bending stiffness has not yet been completed experimentally. The junction between the laser-cut and braided segments exhibits pronounced stress concentration under extreme bending displacement (Figure 7E), and the long-term fatigue behavior of this interconnection, such as potential fretting wear between braided filaments and laser-cut aperture edges under cyclic loading, remains inadequately characterized. Third, the *in vivo* experiments employed standalone 72-filament braided stents rather than the hybrid prototype. Direct chronic implantation of hybrid stent II remains indispensable to confirm junction integrity, foreshortening reduction, and end-constriction elimination under physiological conditions, and is therefore prioritized as the next translational step. Fourth, the *in vitro* test battery (radial compression and three-point bending) primarily assesses structural mechanics but does not directly evaluate clinical deployment performance. Metrics such as microcatheter pushability, trackability through simulated tortuous vasculature, resheathing force across the aneurysm neck, and operator-handling scores were not assessed. Consequently, whether the hybrid design facilitates rapid, smooth, and accurate delivery with minimal resheathing remains to be established through dedicated delivery-system testing and simulated use studies. These acute deployment stresses, together with chronic hemodynamic performance (endothelialization, neointimal coverage, and long-term aneurysm occlusion rates), will be systematically investigated in subsequent preclinical studies.

Future directions therefore include: geometric optimization of the junction (e.g., filleted aperture edges, increased hole diameter, or graded thickness transitions) to further mitigate stress concentration; pulsatile fatigue bench testing and fluid–structure interaction analyses to evaluate the stent under cyclic hemodynamic loading, alongside standardized experimental quantification of bending stiffness to complement the present qualitative flexibility assessment; direct chronic animal implantation of hybrid stent II to validate the integrated device under physiological wall shear stress, drag, and bending conditions; and simulated clinical deployment studies to quantify delivery performance and resheathing characteristics.

## 5 Conclusion

The treatment of complex wide-necked intracranial aneurysms in tortuous cerebrovascular anatomy presents a persistent clinical paradox: monolithic stent designs cannot simultaneously deliver the high radial strength required for distal anchoring and the exceptional flexibility needed to conform to curved vessels without kinking, migration, or incomplete wall apposition. This study addresses this unmet need by proposing a functionally zoned hybrid intracranial stent that heterogeneously integrates a laser-cut anchoring segment with a braided mid-section via an interconnection mechanism wherein braided filaments traverse precision-machined holes.

The optimized single-ended hybrid configuration (hybrid stent II) achieves a mechanically balanced profile that bridges the performance gap between conventional laser-cut and braided stents. Experimentally, it attained an intermediate radial strength of 2.89 N, representing a 36.3% increase over the pure braided stent (2.12 N) while avoiding the excessive rigidity of the laser-cut stent (5.04 N). Its bending stiffness (0.00022 N/mm) remained numerically comparable to braided levels, and the device preserved structural integrity throughout 180° bending—in stark contrast to the pure laser-cut stent, which fractured at 90°. Axial foreshortening was reduced to 18.1%, markedly lower than pure braided configurations (22.0%–22.2%), thereby mitigating the positioning inaccuracies and incomplete aneurysm-neck coverage risks associated with excessive length reduction in tortuous vasculature. Critically, the laser-cut segment’s traction effect completely eliminated the end-constriction phenomenon inherent to standalone braided architectures, ensuring uniform luminal diameter and continuous wall apposition throughout the stent length.

The acute and chronic performance of the braided segment was indirectly validated through 12-month implantation in a rabbit subclavian artery aneurysm model, confirming deliverability through 90° tortuosity, favorable wall apposition, and sustained flow-diversion efficacy. These findings support the functional zoning strategy: the braided section serves as the primary flow-diversion and conformability zone, while the laser-cut end provides distal anchoring to resist migration under cyclic hemodynamic drag and pulsatile wall shear stress.

It is acknowledged that the present study constitutes a structural feasibility and comparative mechanical characterization rather than a comprehensive preclinical validation. Quantitative experimental measurement of bending stiffness, pulsatile fatigue testing of the junction under cyclic loading, and direct chronic implantation of the hybrid prototype to confirm interconnection durability *in vivo* remain essential future steps. Nevertheless, the evidence presented herein—spanning Nitinol-based finite element analysis, standardized *in vitro* mechanical testing, and long-term animal data for the braided therapeutic component—establishes a robust foundation for the hybrid design. This functionally zoned heterogeneous integration strategy provides a promising and mechanically comprehensive endovascular solution for complex intracranial aneurysms in challenging anatomical environments.

## Data Availability

The original contributions presented in the study are included in the article/supplementary material, further inquiries can be directed to the corresponding authors.
